# Aging attenuates diurnal lipid uptake by brown adipose tissue

**DOI:** 10.18632/aging.204318

**Published:** 2022-10-04

**Authors:** Wietse In het Panhuis, Milena Schönke, Ricky Siebeler, Salwa Afkir, Rianne Baelde, Amanda C.M. Pronk, Trea C.M. Streefland, Hetty C.M. Sips, Reshma A. Lalai, Patrick C.N. Rensen, Sander Kooijman

**Affiliations:** 1 Division of Endocrinology, Department of Medicine, Leiden University Medical Center, Leiden, The Netherlands; 2 Einthoven Laboratory for Experimental Vascular Medicine, Leiden University Medical Center, Leiden, The Netherlands

**Keywords:** brown adipose tissue, aging, diurnal rhythm, lipoprotein metabolism, APOE*3-Leiden.CETP mice

## Abstract

Brown adipose tissue (BAT) contributes to cardiometabolic health by taking up glucose and lipids for oxidation, a process that displays a strong diurnal rhythm. While aging has been shown to reduce thermogenic characteristics of BAT, it is as yet unknown whether this reduction is specific to the time of day. Therefore, we assessed whole-body and BAT energy metabolism in young and middle-aged male and female C57BL/6J mice and studied the consequences for lipid metabolism in humanized APOE*3-Leiden.CETP mice (also on a C57BL/6J background). We demonstrate that in middle-aged versus young mice body temperature is lower in both male and female mice, while uptake of triglyceride (TG)-derived fatty acids (FAs) by BAT, reflecting metabolic activity, is attenuated at its peak at the onset of the dark (wakeful) phase in female mice. This coincided with delayed plasma clearance of TG-rich lipoproteins and TG-depleted lipoprotein core remnants, and elevated plasma TGs at the same time point. Furthermore, middle-aged female mice showed increased adiposity, accompanied by lipid accumulation, increased expression of genes involved in lipogenesis, and reduced expression of genes involved in fat oxidation and the intracellular clock machinery in BAT. Peak abundance of lipoprotein lipase (LPL), a crucial regulator of FA uptake, was attenuated in BAT. Our findings suggest that LPL is a potential therapeutic target for restoring diurnal metabolic BAT activity, and that efficiency of strategies targeting BAT may be improved by including time of day as an important factor.

## INTRODUCTION

Aging gradually increases the risk of cardiometabolic diseases through – among others – increased adiposity and elevated plasma lipid levels, starting already in young adulthood [1]. Although the etiology is likely to be multifactorial, an important contributing process is adipose tissue remodeling [2]. Besides an overall increase in adiposity, aging is characterized by higher visceral vs. subcutaneous white adipose tissue (WAT) ratio, and lower thermogenic activity of brown adipose tissue (BAT) [3–5].

Whilst WAT stores nutritional energy as triglycerides (TGs), BAT takes up large amounts of glucose and TG-derived fatty acids (FAs) for oxidation to produce heat. Accordingly, nutrient uptake by BAT can be used as a proxy for its thermogenic activity [6–9], and many favorable cardiometabolic effects have been attributed to BAT activation in mice [10, 11]. The presence of metabolically active BAT in humans is also associated with cardiometabolic health [12, 13], though more prospective studies are needed to establish the causality of this relationship. Interestingly, we and others have demonstrated that both glucose and lipid uptake by BAT is characterized by a high amplitude diurnal oscillation (i.e., rhythms synchronized to a light-dark cycle) [14–19], likely to maintain body temperature during the rest phase and facilitate the rise in body temperature prior to wakening.

Reduced nutrient uptake by BAT may (partly) explain the age-related increase in adiposity and elevated plasma lipid levels. It is, however, unknown if aging causes an overall reduction in nutrient uptake by BAT or a time of day-specific reduction, and whether this has consequences for diurnal rhythms of plasma (postprandial) lipid levels. Insight into age-related changes in diurnal nutrient uptake by BAT may lead to the identification or optimization of strategies to promote nutrient uptake by BAT and improve cardiometabolic health.

In this study, we aimed to assess the consequences of aging on diurnal nutrient uptake in BAT and show that in middle-aged mice lipid uptake is attenuated at its diurnal peak in female but not male C57BL/6J mice, which coincided with elevated plasma lipid levels and increased adiposity in APOE*3-Leiden.CETP mice (on a C57BL/6J background), a well-established mouse model for human lipoprotein metabolism [20, 21].

## RESULTS

### Aging attenuates body temperature rhythm in male and female wildtype mice, but decreases uptake of lipids by brown adipose tissue in females only

**Figure 1:**
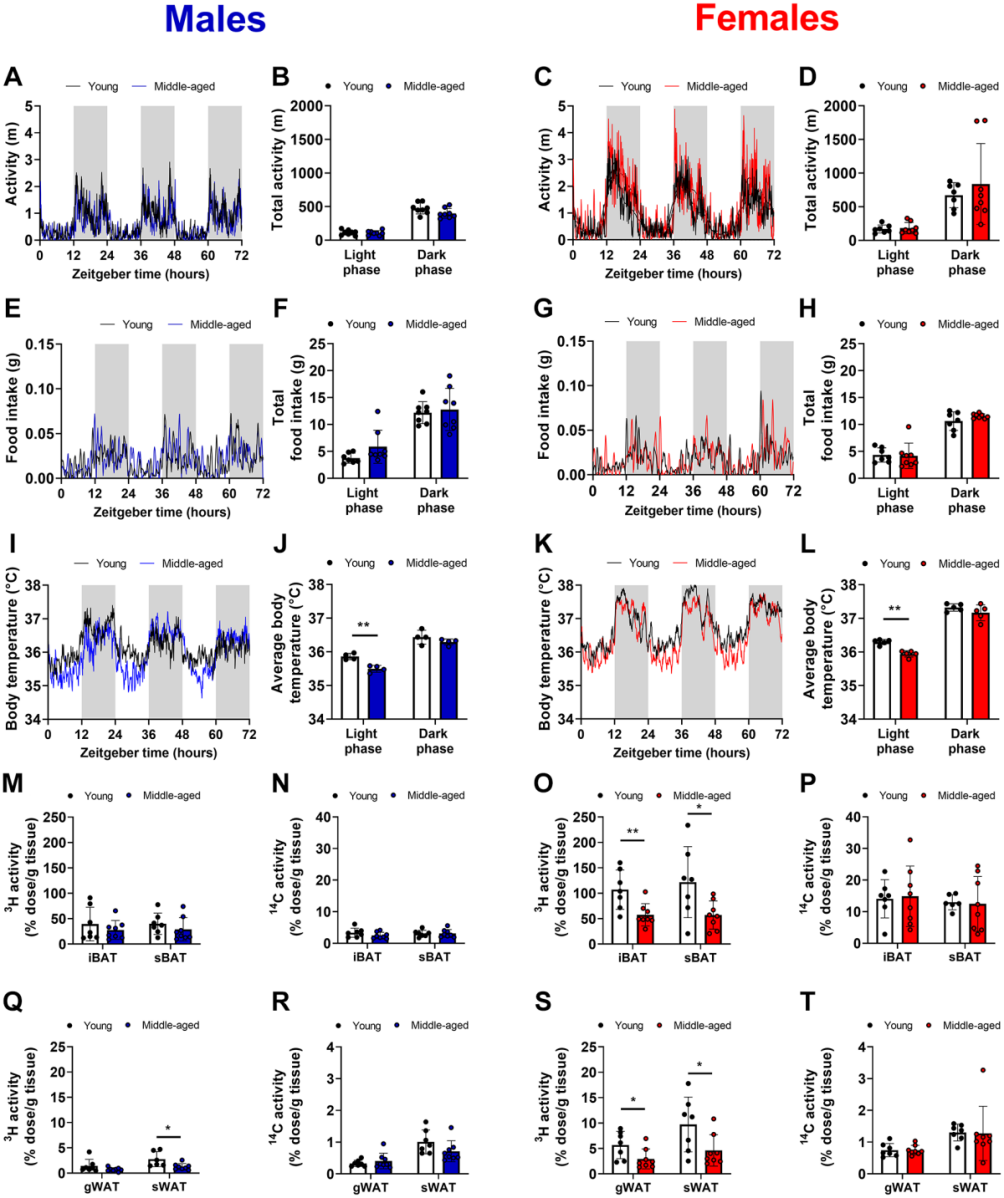
**Voluntary activity, food intake, core body temperature, and triglyceride-derived fatty acid uptake by brown and white adipose tissue during aging.** Young (12 weeks old) and middle-aged (52 weeks old) male (left panels in blue) and female (right panels in red) C57BL/6J mice were single-housed in metabolic home-cages for continuous measurement of (**A**–**D**) voluntary locomotor activity (*n* = 7–8 mice/group), (**E**–**H**) food intake (*n* = 7–8 mice/group), and (**I**–**L**) core body temperature with implanted telemetric probes (*n* = 5 mice/group). Mice were injected with triglyceride-rich lipoprotein-like particles labeled with glycerol tri[^3^H]oleate and 2-[1-^14^C]-deoxyglucose at *Zeitgeber* Time 12 to assess uptake of the radiolabels by interscapular brown adipose tissue (iBAT) and subscapular BAT (sBAT) (**M**–**P**) and by gonadal white adipose tissue (gWAT) and subcutaneous WAT (sWAT) (**Q**–**T**) (*n* = 6–8 mice/group). Bar graphs represent means ± SD. ^*^p < 0.05; ^**^p < 0.01, according to unpaired *t*-test or two-way ANOVA and following Šídák's multiple-comparison test.

To study the metabolic consequences of aging, young (12 weeks old) and middle-aged (52 weeks old) male and female wildtype mice (C57BL/6J background) were utilized. In both sexes, middle-aged mice had a higher body weight (Supplementary Figure 1A, 1C) than young mice explained by higher lean mass (Supplementary Figure 1B, 1D) and fat mass (Supplementary Figure 1E, 1G), though with an increased body fat percentage (Supplementary Figure 1F, 1H). To evaluate the effects of aging on energy homeostasis; energy intake and energy expenditure (indirect calorimetry, voluntary activity in home cage, and core body temperature) were monitored for 3 days. Despite differences in body weight, there was no difference in voluntary activity (Figure 1A–1D), food intake (Figure 1E–1H), energy expenditure estimated from oxygen consumption and carbon dioxide production (Supplementary Figure 2A–2D) and respiratory exchange ratio (Supplementary Figure 2E–2H) between young and middle-aged mice in both sexes, indicating that middle-aged mice might have an altered substrate partitioning. Indeed, core body temperature was reduced during the light (inactive) phase in middle-aged mice of both sexes (Figure 1I–1L). To assess if this effect could be attributed to altered metabolic activity in adipose tissue, we intravenously injected mice with TG-rich lipoprotein-like particles labeled with glycerol tri[^3^H]oleate, and 2-[1-^14^C]-deoxyglucose at the end of the light phase, denoted as *Zeitgeber* Time (ZT)12. This time point also corresponds to the previously reported peak in metabolic BAT activity [17], and changes in BAT activity at this time of day are therefore expected to have the biggest metabolic impact. In line with previous experiments, uptake of both radiolabels was higher in BAT than in WAT independent of sex (Figure 1M–1T), and BAT showed higher metabolic activity in females than in males regardless of age, although we could not formally test as the sexes were treated as separate cohorts. In middle-aged male mice, [^3^H]oleate uptake by interscapular (i)BAT and subscapular (s)BAT was non-significantly reduced when compared to young male mice (Figure 1M). Uptake of [^3^H]oleate by subcutaneous (s)WAT but not gonadal (g)WAT was lower in middle-aged male mice compared with young male mice. In middle-aged female mice, [^3^H]oleate uptake by the BAT depots was significantly lower, and also uptake by sWAT and gWAT was lower compared with young mice (Figure 1O). Interestingly, 2-[1-^14^C]-deoxyglucose uptake by BAT and WAT was unchanged between young and middle-aged mice of both sexes (Figure 1N, 1P, 1R, 1T), illustrating tissue and substrate-specific alterations in nutrient handling during aging. Collectively, these data show that specifically in females aging decreases the uptake of lipids by BAT as measured at the onset of the dark phase.

### Aging attenuates processing of triglyceride-rich lipoproteins in APOE*3-Leiden.CETP mice at peak activity of brown adipose tissue

To determine whether the reduction in TG-derived FA uptake by BAT and WAT in aged female mice is caused by a time of day-specific decrease or an overall decrease in metabolic activity of the tissue, we performed a new study comparing lipid handling at the end of the light phase (ZT12) and dark phase (ZT0), corresponding to peak and nadir TG-derived FA uptake by BAT [17], as a proxy for diurnal uptake. This time we utilized female APOE*3-Leiden.CETP mice (also on a C57BL/6J background), which is a well-established mouse model for human lipoprotein metabolism, to simultaneously address lipid uptake by BAT and the clearance of TG-depleted lipoprotein core remnants by the liver. Body weight and composition were monitored over 16 weeks while the mice were fed a cholesterol-enriched Western-type diet (25% kJ from fat) to induce hyperlipidemia, starting at the age of 11–15 weeks in young mice and 51–55 weeks in middle-aged mice. Throughout this period, compared to young mice, middle-aged mice consistently showed higher body weight (Supplementary Figure 3A) due to higher lean mass (Supplementary Figure 3B) and fat mass (Supplementary Figure 3C), as well as a higher body fat percentage (Supplementary Figure 3D). Food intake was higher in middle-aged mice than young mice (Supplementary Figure 3E), but lower when expressed relative to body weight (Supplementary Figure 3F), and unchanged when adjusted for lean mass (Supplementary Figure 3G).

**Figure 2:**
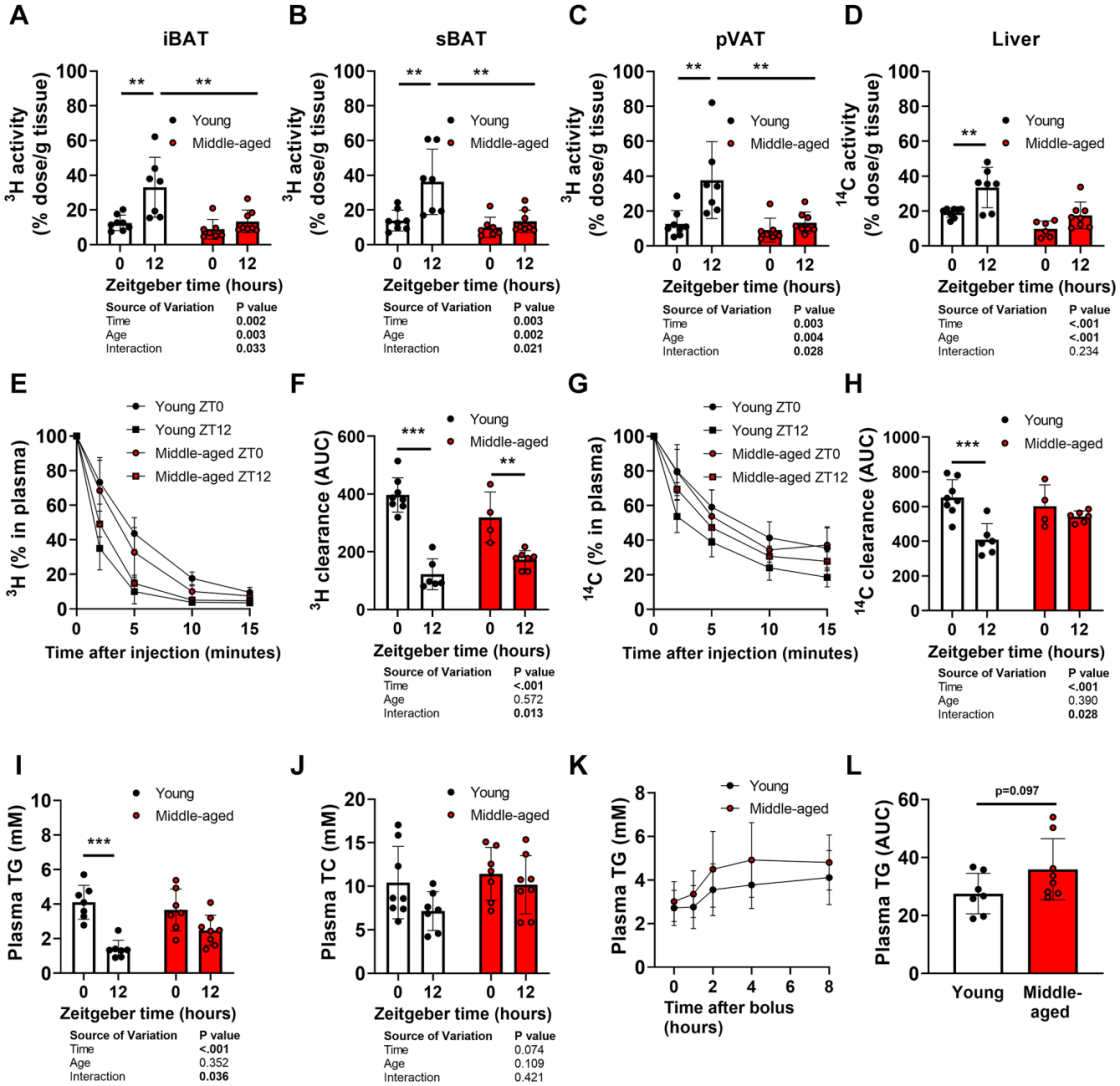
**Oscillations of organ uptake, plasma clearance, and plasma levels of lipids during aging.** Young (27–31 weeks old) and middle-aged (67–71 weeks old) female APOE*3-Leiden.CETP mice were injected with triglyceride (TG)-rich lipoprotein-like particles double-labeled with glycerol tri[^3^H]oleate and [^14^C]cholesteryl oleate at *Zeitgeber* Time 0 and 12 (*n* = 7–8 mice/group/time point) to assess [^3^H]oleate uptake by (**A**) interscapular brown adipose tissue (iBAT), (**B**) subscapular BAT (sBAT), and (**C**) perivascular adipose tissue (pVAT), and (**D**) [^14^C]cholesteryl oleate uptake by the liver, and plasma decay of (**E**, **F**) [^3^H]oleate and (**G**, **H**) [^14^C]cholesteryl oleate (*n* = 4–8 mice/group/time point). Fasted plasma levels of (**I**) TG and (**J**) total cholesterol (TC) (*n* = 7–8 mice/group/time point). (**K**) Oral lipid tolerance test performed at ZT12 with blood collection for plasma TG measurement, after which (**L**) the area under the curve (AUC) was calculated (*n* = 7–8 mice/group). Bar graphs and data points on curves represent means ± SD. ^*^p < 0.05; ^**^p < 0.01; ^***^p < 0.001, according to unpaired *t*-test or two-way ANOVA and following Tukey’s multiple-comparison test.

At the endpoint, plasma clearance and organ uptake of glycerol tri[^3^H]oleate and [^14^C]cholesteryl oleate from intravenously injected TG-rich lipoprotein-like particles were assessed. In WAT and skeletal muscles (i.e., extensor digitorum longus, tibialis anterior, and soleus), [^3^H]oleate uptake, reflecting TG-derived FA uptake, was not different between ZT12 and ZT0 in young mice and lowered in middle-aged mice (Supplementary Figure 4). In contrast, [^3^H]oleate uptake by iBAT, sBAT, and perivascular adipose tissue (pVAT) was 3-fold higher at ZT12 compared with ZT0 in young mice, while [^3^H]oleate uptake in middle-aged mice was not oscillating (Figure 2A–2C). Notably, [^3^H]oleate uptake by the BAT depots in the older mice was not different from the anticipated nadir at ZT0 in young mice (Figure 2A–2C), indicating a time of day-specific reduction rather than a total reduction in activity (time-age interaction by two-way ANOVA: *P* = 0.033, *P* = 0.021, and *P* = 0.028 for respectively iBAT, sBAT, and pVAT). [^14^C]cholesteryl oleate uptake by the liver, reflecting the clearance of delipidated lipoprotein remnants, was higher at ZT12 compared with ZT0 in young mice, but not in middle-aged mice. Interestingly, [^14^C]cholesteryl oleate uptake by the liver was decreased by age, regardless of time of day (age effect by two-way ANOVA: *P* < 0.001) (Figure 2D).

In line with the higher TG-derived FA uptake by BAT and hepatic uptake of TRL-remnants at ZT12 compared to ZT0 in young mice, plasma decay of [^3^H]- and [^14^C]-derived activity was faster at ZT12 than at ZT0 in young mice (Figure 2E–2H). In contrast, these differences were attenuated in middle-aged mice (time-age interaction by two-way ANOVA: *P* = 0.013 and *P* = 0.028 for respectively [^3^H]oleate and [^14^C]cholesteryl oleate decay; significant differences between groups at individual time points not shown). The diurnal variation in the ability to clear plasma lipids was accompanied by a diurnal variation in plasma TG levels in young, but not middle-aged mice (Figure 2I). Total cholesterol levels were not different at ZT12 vs. ZT0 regardless of age (Figure 2J). Postprandial TG excursions following an oral lipid tolerance test with a fixed amount of olive oil at ZT12 tended to be elevated in middle-aged mice (*P* = 0.097; Figure 2K, 2L).

### Aging promotes whitening of brown adipose tissue in female APOE*3-Leiden.CETP mice

**Figure 3:**
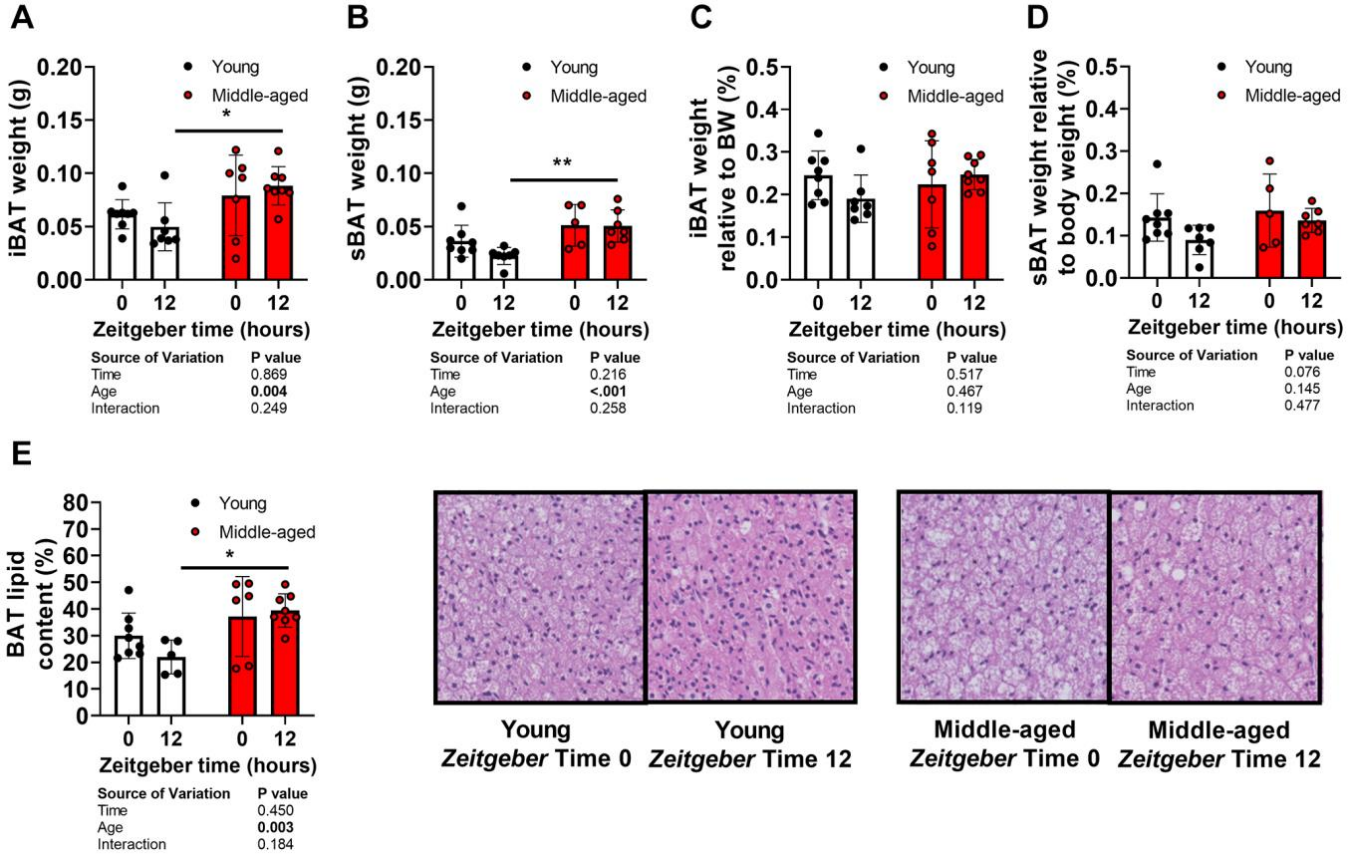
**Brown adipose tissue morphology and aging.** Tissues of young (27–31 weeks old; white circles) and middle-aged (67–71 weeks old; black circles) female APOE*3-Leiden.CETP mice were collected at *Zeitgeber* Time 0 and 12 (*n* = 7–8 mice/group/time point). (**A**) interscapular brown adipose tissue (iBAT) and (**B**) subscapular BAT (sBAT) were weighed, and (**C**, **D**) expressed as percentage of body weight (*n* = 5–8 mice/group/time point). (**E**) Lipid content of sBAT was quantified and representative pictures are shown (*n* = 5–8/group/time point). Bar graphs represent means ± SD. ^*^p < 0.05; ^**^p < 0.01, according to two-way ANOVA and following Tukey’s multiple-comparison test.

To investigate mechanisms underlying time of day-specific reductions in TG-derived FA uptake by BAT during aging, morphology and markers of BAT function in tissues collected from female APOE*3-Leiden.CETP mice at ZT0 and ZT12 were analyzed. Middle-aged mice showed greater iBAT (Figure 3A) and sBAT (Figure 3B) mass compared with young mice, but not when expressed relative to body weight (Figure 3C, 3D). In line with a higher body fat percentage, histological analysis of lipid content of sBAT showed increases in middle-aged mice (age effect by two-way ANOVA: *P* = 0.003) (Figure 3E), indicating whitening of the tissue.

**Figure 4:**
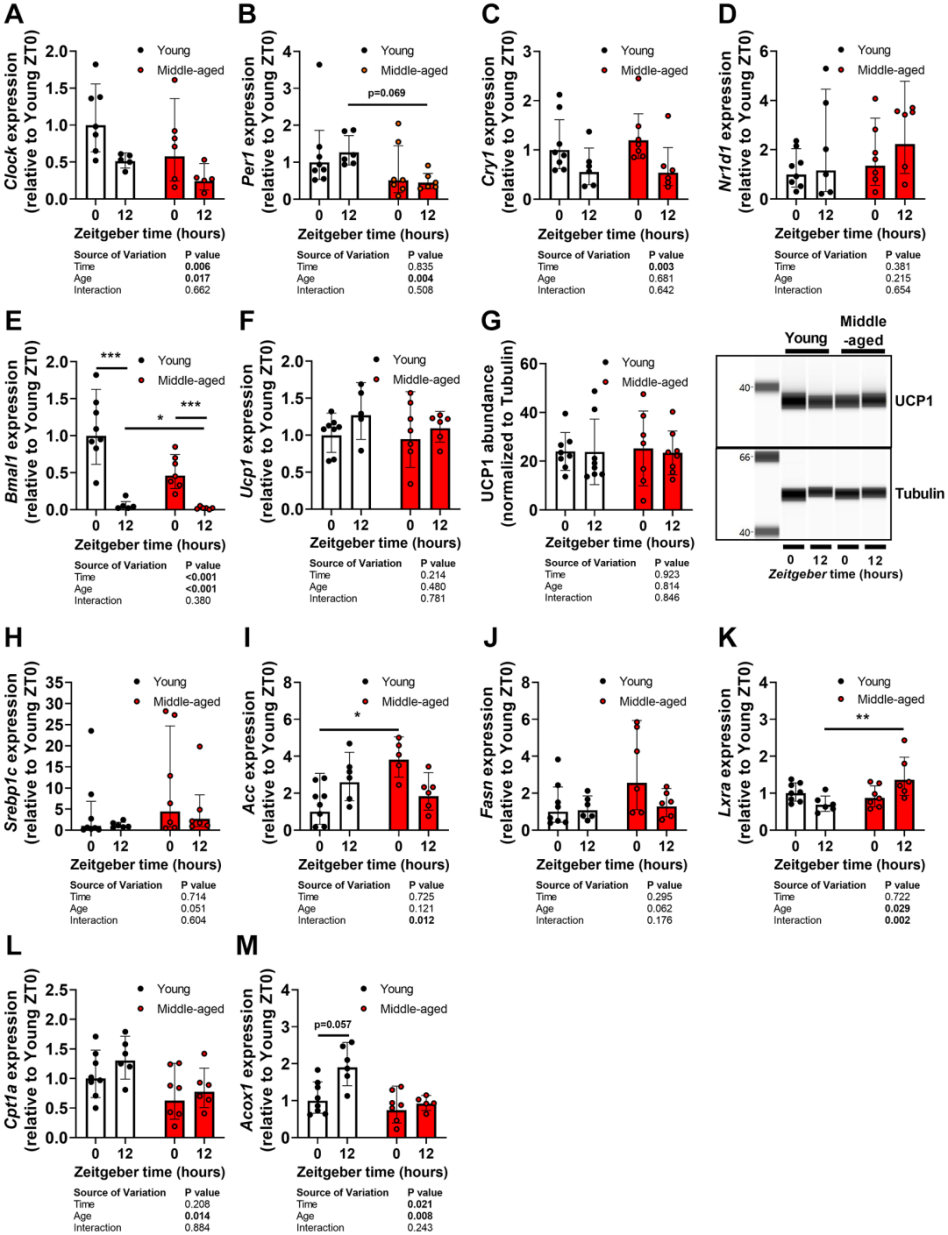
**Lipogenesis, beta-oxidation, and the cellular clock in brown adipose tissue during aging.** Subscapular brown adipose tissue of young (27–31 weeks old; white circles or bars) and middle-aged (67–71 weeks old; black circles or bars) female APOE*3-Leiden.CETP mice was collected at *Zeitgeber* Time 0 and 12 (*n* = 7–8 mice/group/time point). Gene expression of (**A**) circadian locomotor output cycles kaput (*Clock*), (**B**) period 1 (*Per1*), (**C**) cryptochrome 1 (*Cry1*), (**D**) *Nr1d1* (REV-ERBA), (**E**) brain and muscle ARNT-like 1 (*Bmal1*), and (**F**) uncoupling protein 1 (*Ucp1*) as determined by quantitative polymerase chain reaction, normalized to *ß-actin*, and shown relative to the expression in young mice at ZT0 (*n* = 5-8 mice/group/time point). (**G**) Protein abundance of UCP1 as measured by automated Western blotting, and normalized to Tubulin levels (*n* = 7–8 mice/group/time point). Gene expression of (**H**) sterol regulatory element-binding protein 1c (*Srebp1c*), (**I**) acetyl-CoA carboxylase (*Acc*), (**J**) fatty acid synthase (*Fasn*), (**K**) liver X receptor alpha (*Lxra*), (**L**) carnitine palmitoyltransferase 1a (*Cpt1a*), and (**M**) peroxisomal acyl-coenzyme A oxidase 1 (*Acox1*). Bar graphs represent means ± SD. ^*^p < 0.05; ^**^p < 0.01, ^***^p < 0.001, according to two-way ANOVA and following Tukey’s multiple-comparison test.

To investigate if attenuated diurnal BAT activity during aging coincides with changes in the cellular clock machinery, a major driving force behind cellular oscillations, expression of several clock genes was measured in BAT. Whilst patterns for time-dependent expression of circadian locomotor output cycles kaput (*Clock*), period 1 (*Per1*), cryptochrome 1 (*Cry1*), nuclear receptor subfamily 1 group D member 1 (*Nr1d1*, encoding REV-ERBA), and brain and muscle ARNT-like 1 (*Bmal1*) were not different (Figure 4A–4E), middle-aged mice showed reduced expression of *Clock*, *Per1*, and *Bmal1* when compared to young mice (age effect by two-way ANOVA: *P* = 0.017, *P* = 0.004, and *P* < 0.001, for respectively *Clock*, *Per1*, and *Bmal1*).

Next, we assessed markers of thermogenesis, lipogenesis, and beta oxidation in the same tissues. Gene expression (Figure 4F) and protein abundance (Figure 4G) of uncoupling protein 1 (UCP1) were not different between ZT0 and ZT12, or affected by age. Expression of lipogenic genes sterol regulatory element-binding protein 1c (*Srebp1c*) (Figure 4H), acetyl-CoA carboxylase (*Acc*) (Figure 4I), and fatty acid synthase (*Fasn*) (Figure 4J) tended to be increased in middle-aged mice (age effect by two-way ANOVA: *P* = 0.051, *P* = 0.121, and *P* = 0.062, for *Srebp1c*, *Acc*, and *Fasn*, respectively). In addition, expression of liver X receptor alpha (*Lxra*) (Figure 4K), a negative regulator of beta-oxidation in BAT [22], was significantly increased at ZT12 in middle-aged mice compared with young mice. Accordingly, gene expression of *Cpt1a* (Figure 4L), encoding a crucial factor for FA transport into mitochondria prior to beta oxidation [23], and peroxisomal acyl-coenzyme A oxidase 1 (*Acox1*) (Figure 4M), encoding an enzyme involved in beta oxidation, was reduced in middle-aged mice (age effect by two-way ANOVA: *P* = 0.014 and *P* = 0.008, for respectively *Cpt1a* and *Acox1*). These data suggest altered functioning of BAT with an increase in lipogenesis and reduction in fat oxidation, but do not explain the attenuated lipid uptake by the tissue.

### Aging attenuates lipoprotein lipase expression and abundance in brown adipose tissue of female APOE*3-Leiden.CETP mice

**Figure 5:**
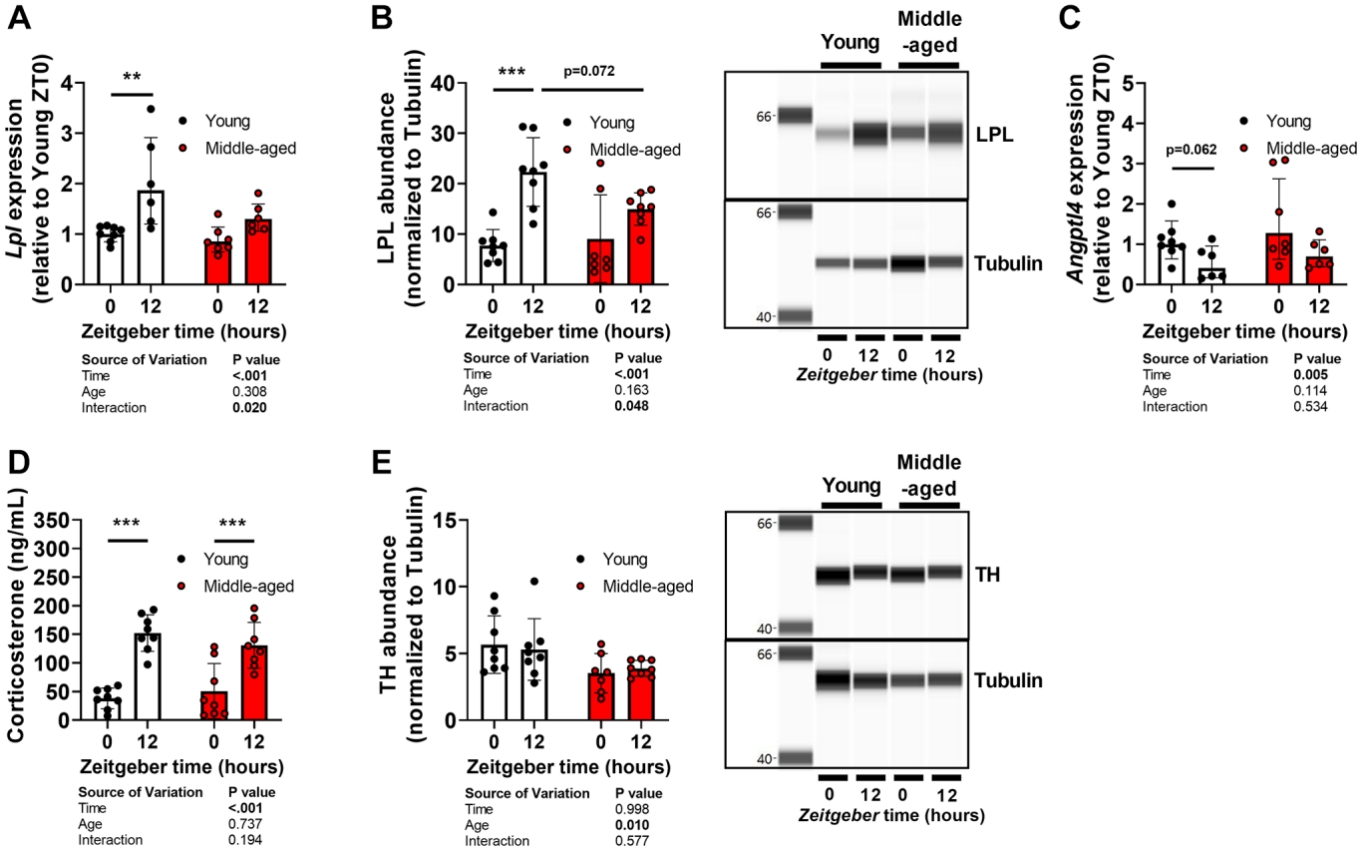
**Oscillations of lipoprotein in brown adipose tissue and its regulators during aging.** Subscapular brown adipose tissue of young (27–31 weeks old; white circles or bars) and middle-aged (67–71 weeks old; black circles or bars) female APOE*3-Leiden.CETP mice was collected at *Zeitgeber* Time 0 and 12 (*n* = 7–8 mice/group/time point). (**A**) Gene expression of lipoprotein lipase (*Lpl*) measured by quantitative polymerase chain reaction, normalized to *ß-actin*, and shown relative to the expression in young mice at ZT0. (**B**) Protein abundance of LPL measured by automated Western blot, and normalized to Tubulin levels. (**C**) Gene expression of angiopoietin-like 4 (*Angptl4*). (**D**) Corticosterone levels measured in plasma collected from young (17–21 weeks old) and middle-aged (57–61 weeks old) mice at *Zeitgeber* Time 0 and 12. (**E**) Protein abundance of tyrosine hydroxylase (TH). Bar graphs represent means ± SD. ^**^p < 0.01; ^***^p < 0.001, according to two-way ANOVA and following Tukey’s multiple-comparison test.

We next assessed expression of *Lpl* and its negative regulator *Angptl4* in BAT, as we have previously shown that these are crucial regulators of diurnal TG-derived FA uptake [17, 24]. *Lpl* gene expression (Figure 5A) and LPL protein abundance (Figure 5B) were higher at ZT12 than ZT0 in young mice, in line with previous observations [17, 24], but not in middle-aged mice. *Angptl4* expression tended to be lower at ZT12 than ZT0 regardless of age (Figure 5C).

We previously also demonstrated involvement of glucocorticoids and the sympathetic nervous system in the diurnal control of BAT activity including the regulation of LPL and ANGPTL4 activity [14, 17]. However, corticosterone levels were not different between young and middle-aged mice (Figure 5D). Protein abundance of tyrosine hydroxylase (TH) (Figure 5E), the rate-limiting enzyme in the synthesis of noradrenalin and a marker of sympathetic activity, was reduced in middle-aged mice (age effect by two-way ANOVA: *P* = 0.012). However, TH abundance was not different between ZT0 and ZT12 in young or middle-aged mice.

## DISCUSSION

BAT displays pronounced diurnal variation in transcriptional activity, lipid content, and nutrient uptake [17, 18, 24, 25], of which the latter highly contributes to the beneficial effects of BAT on cardiometabolic health [10, 11]. While previous studies show an age-related dampening in oscillations of mRNA levels and lipid abundance in BAT [25, 26], it is unknown how aging affects diurnal nutrient uptake by BAT. Here we demonstrated that with age this rhythm of nutrient uptake is attenuated characterized by a selective reduction in TG-derived FA uptake at the onset of the dark phase in specifically female mice, an effect that was accompanied by elevated plasma lipid levels and by adipose tissue remodeling.

In females, metabolic activity of BAT is generally higher than in males probably because females can rely to a lesser extent on thermogenesis by skeletal muscle [27]. The age-related reduction in TG-derived FA uptake may therefore be more pronounced and picked up at an earlier age in females compared to males. This is likely because previous studies have reported overall reductions in thermogenic characteristics based on glucose uptake, morphology, and mRNA and protein levels in BAT [3–5] in both sexes at far higher age of 2 years and older. Similarly, the fact that we studied middle-aged rather than old mice may also explain why uptake of TG-derived FAs, but not glucose, was decreased in middle-aged female C57BL/6J mice, since aging has often been reported to reduce glucose uptake by BAT in mice and humans [3, 6, 28]. As thermogenesis in BAT is particularly dependent on FA oxidation rather than glucose oxidation [29, 30], it is possible that reductions in TG-derived FA uptake precede reductions in glucose uptake during aging.

We subsequently assessed the consequences of attenuated TG-derived FA uptake by BAT on lipoprotein metabolism in Western-type diet-fed APOE*3-Leiden.CETP mice. To be able to do so independent of the age-related changes in very-low-density-lipoprotein-cholesterol secretion from the liver [31], we decided to increase the cholesterol content of the diet for the middle-aged APOE*3-Leiden.CETP mice so that plasma cholesterol levels were comparable between groups. The relatively low fat content (25 kJ% fat) is not expected to induce altered circadian rhythms as reported for classical high fat diets (45-60 kJ% fat), but we cannot fully exclude an age-diet interaction. In this context, we observed that attenuated TG-derived FA uptake by BAT at the onset of the dark phase, corresponding with the peak in daily activity, resulted in elevated plasma (postprandial) TG levels and reduced hepatic uptake of TRL-remnants. Involvement of other metabolic tissues in the attenuated ZT0-ZT12 differences in lipoprotein metabolism cannot be excluded, but may be limited as BAT is the metabolic organ with the most pronounced diurnal oscillations in lipid uptake [17]. These observations in mice are very comparable to what we and others have previously reported for humans, with dampened rhythms of plasma lipid levels and impaired postprandial lipid handling in the early wake phase in aged individuals [1, 32, 33]. We anticipate that the de-regulation of these oscillations impairs cardiometabolic health [34, 35].

Mechanistically, aging caused a time-age interaction, with a trend for lower LPL abundance at ZT12, a factor which we previously identified as crucial regulator of diurnal metabolic BAT activity [17, 24] driven by rhythmic activity of glucocorticoids and possibly the sympathetic nervous system [14, 36]. In the current study we did not find evidence for altered glucocorticoid levels or oscillations; an effect that is expected at higher age in C57BL/6J mice [37, 38]. We observed a reduction in abundance of sympathetic activity marker TH, although this should be interpreted with caution as it was not significantly different between ZT0 and ZT12 in young mice. Previous findings showed attenuations in both oscillations of glucocorticoids and sympathetic stimulation and signaling in BAT in mice and humans during aging [39–41], indicating that these factors may influence diurnal BAT activity at older ages. Interestingly, we did observe reduced expression of clock genes in BAT of mice at middle-age. Regular expression of clock genes is important for metabolic health as mutations in humans and knockout in mice promote cardiometabolic disease [42]. In line with our observations that middle-aged mice show reduced *Bmal1* expression, loss of *Bmal1* in BAT has been shown to result in reduced beta oxidation and to promote whitening of BAT [43]. Given that nearly half of the transcriptome of BAT is oscillating with distinctive expression patterns [24], future studies should include more time points to delineate mechanisms underlying the age-related attenuation of diurnal metabolic BAT activity.

The million-dollar question is how to prevent or reverse aging-associated diseases. Several strategies have shown to be effective in improving the age-related decline in total BAT activity, such as exercise [44], caloric restriction [45], cold exposure [46], and orexin [47]. Our observation that aging reduces lipid uptake by BAT particularly at its peak suggests that efficiency of strategies targeting BAT can be improved by including time of day as an important factor. We additionally postulate LPL as a potential therapeutic target for restoring diurnal metabolic BAT activity from middle-age onwards, and various novel strategies to regulate LPL expression and activity are currently under development [48].

## METHODS

### Animals

All experiments were performed in accordance with the Institute for Laboratory Animal Research Guide for the Care and Use of Laboratory Animals and were approved by the National Committee for Animal experiments. All mice were housed in groups of 2–4 mice per cage under standard conditions with 12:12 hour light-dark cycles at 21°C with *ad libitum* access to water and diet, unless indicated otherwise. In the first experiment, young (12 weeks old) and middle-aged (52 weeks old) male C57BL/6JRj (Janvier Labs, Le Genest-Saint-Isle, France) and female C57BL/6J (Charles River Laboratories, Wilmington, MA, USA) (*n* = 8 mice per group) were fed a chow diet (Rat and Mouse No.3 Breeding; SDS, Horley, United Kingdom) and 4–5 mice from each group were subcutaneously implanted a telemetry transmitter (Sable Systems GmbH, Berlin, Germany) to measure body temperature. After 3 weeks, mice were killed at the onset of the dark phase (ZT12) to assess organ uptake of TG-derived FAs and glucose (see below in the section ‘Clearance of radiolabeled TG-rich lipoprotein-like emulsion particles’). In a second experiment, young (9–13 weeks old) and middle-aged (49–53 weeks old) female APOE*3-Leiden.CETP mice were utilized (*n* = 16 mice per group). APOE*3-Leiden.CETP mice were generated as previously described [21]. Young mice received a diet enriched with 0.10% cholesterol and middle-aged mice a diet enriched with 0.30% cholesterol (both containing 16% fat; Diet T; Ssniff-Spezialdiäten GmbH, Soest, Germany). After a dietary run-in period of 3 weeks, baseline measurements were taken and the animals were followed for 16 weeks to monitor body weight and body composition (EchoMRI 100-Analyzer; EchoMRI, Houston, TX, USA). At week 6, stress-minimized blood was collected in half of the mice at the onset of the light phase (ZT0) and the onset of the dark phase (ZT12) by collecting blood within 2 minutes after lifting the cage to measure plasma corticosterone. At week 8, half of the mice were subjected to a postprandial lipid tolerance test at ZT12. At the end of the study, half of the young (27–31 weeks old) and middle-aged (67–71 weeks old) mice in each group was killed at ZT0 and the other half at ZT12 to assess plasma lipid levels as well as plasma decay and organ uptake of TG-derived FAs and TRL-remnants.

### Body temperature and indirect calorimetry

A telemetric transmitter (weight, 1.1 g; volume, 0.52 mL; G2 E-mitter, Starr, Elst, The Netherlands) was implanted into the abdominal cavity after which the mice were allowed to recover for 2 weeks before they were individually housed in automated metabolic home cages (Promethion System; Sable Systems, Las Vegas, NV, USA) for another 5 days. The first 2 days were considered as acclimatization phase and were not included in the data analysis. Voluntary locomotor behavior (by infrared beam breaks), food intake, body temperature, O_2_ consumption, and CO_2_ production were continuously collected in 5 minute bins and energy expenditure and respiratory exchange ratio were calculated. Energy expenditure was not normalized to lean body mass.

### Clearance of radiolabeled TG-rich lipoprotein-like emulsion particles

Mice received an intravenous injection with an emulsion of TRL-like particles (80 nm) containing glycerol tri[^3^H]oleate (1 mg TG in 200 μL saline per mouse), prepared as described previously [49]. In experiment 1, 2-[1-^14^C]-deoxyglucose (NEC495A250UC; PerkinElmer, Waltham, MA, USA) was added to the emulsion to assess uptake of glucose and in experiment 2, TRL-like particles were double-labelled with [^14^C]cholesteryl oleate in addition to glycerol tri[^3^H]oleate to allow for TRL-remnant clearance. Blood was collected to determine plasma decay of radiolabels. After 15 minutes, mice were killed by CO_2_ inhalation and perfused via the heart with ice-cold PBS. Various organs were collected and dissolved in 0.5 mL Solvable (6NE9100, PerkinElmer, Waltham, MA, USA) at 56°C overnight, after which 5.0 mL Ultima Gold (6013329, PerkinElmer, Waltham, MA, USA) was added. Plasma was directly added to 2.5 mL Ultima Gold. ^3^H-activity and ^14^C-activity were measured with a scintillation counter (Tri-Carb 2910 TR, PerkinElmer, Waltham, MA, USA) and expressed as a percentage of injected dose per gram tissue or as a percentage of injected dose in plasma. Mice were excluded from the calculation of the AUC (between 2 and 15 minutes after injection) of plasma decay if one or more plasma samples were missing.

### Plasma measurements

Plasma corticosterone levels were measured with Corticosterone HS (High Sensitivity) enzyme immunoassay kit (AC-15F1; Immunodiagnostic Systems Holdings Ltd, Boldon, UK), according to the manufacturer’s protocols. Plasma TG and TC levels were measured in plasma collected at week 16 by using Cobas Triglycerides (106571) and Cobas Total Cholesterol (106570) enzymatic kits (both from Roche Diagnostics, Mannheim, Germany), by adding 200 μL reagent (undiluted for TG and 3× diluted for TC) to 2.5 μL sample and incubating at room temperature for 30 min prior to measuring at 492 nm versus 650 nm (for TG) or at 505 nm versus 650 nm (for TC).

### Postprandial lipid tolerance

The animals were fasted for 4 hours prior to receiving an olive oil bolus (200 μL per mouse) (Carbonell, Cordoba, Spain) by oral gavage. Tail vein blood was sampled prior to and 1, 2, 4, and 8 hours post-gavage to measure plasma TG levels.

### Histology

Paraffin-embedded sBAT (approx. 5–10 mg) from mice of experiment 2 was cross-sectioned (5 μm) and stained with Mayer’s hematoxylin (109249, Sigma Aldrich, Saint Louis, MO, USA) and eosin using standard protocols [50]. Lipid content was assessed by quantifying the unstained areas representing intracellular lipid vacuoles using ImageJ software, version 1.52a (National Institutes of Health, Bethesda, MD, USA).

### Protein quantification

Frozen sBAT samples (approx. 5 mg) from mice of experiment 2 were lysed in RIPA buffer (150 mM sodium chloride, 1.0% Triton X-100, 0.5% sodium deoxycholate, 0.1% sodium dodecyl sulphate, 50 mM Tris pH 8.0, protease and phosphatase inhibitors (A32959, Thermo Fisher Scientific, Waltham, MA, USA), homogenized by a FastPrep-24™ 5G bead beating grinder and lysis system (4.0 m/s for 10 sec; MP Biomedicals™, Santa Ana, CA, USA) and centrifuged repeatedly (16.2 *g* for 5 min at 4°C) to remove fat. Protein concentrations were determined using the Pierce™ BCA Protein Assay Kit (23225, Thermo Fisher Scientific, Waltham, MA, USA), according to manufacturer’s protocol. Protein abundance was assessed by automated Western blot using Wes™ (ProteinSimple, Santa Clara, CA, USA) using a rabbit anti-mouse UCP1 antibody (1:20; U6382, Sigma Aldrich, Saint Louis, MO, USA), a goat anti-mouse LPL antibody (1:50; kind gift from André Bensadoun [51]), and a rabbit anti-mouse TH antibody (1:50; ab137869, Abcam, Cambridge, United Kingdom). A rabbit anti-mouse Tubulin antibody (1:10; 2148, Cell Signaling, Danvers, MA, USA) was used for normalization. Protein lysates of 0.02, 0.80, 0.20, and 0.80 μg/μL were used for UCP1, LPL, TH, and Tubulin, respectively. An anti-goat antibody (DM-006, ProteinSimple, Santa Clara, CA, USA) was used for LPL and an anti-rabbit antibody (DM-001, ProteinSimple, Santa Clara, CA, USA) was used for UCP1, TH, and Tubulin. Relative normalized protein levels were quantified by Compass software (ProteinSimple; v5.0.1).

### Gene expression analysis

RNA was isolated from frozen sBAT (approx. 5 mg) from mice of experiment 2 by lysing and homogenization using TriPure RNA Isolation Reagent (11667165001, Sigma Aldrich, Saint Louis, MO, USA) and a FastPrep-24™ 5G bead beating grinder and lysis system (4.0 m/s for 10 sec; MP Biomedicals™, Santa Ana, CA, USA). cDNA was synthesized from 1 μg RNA using M-MLV Reverse Transcriptase (M1705, Promega, Madison, WI, USA) and qPCR was conducted utilizing SYBR green kit (Promega, Madison, WI, USA) and a CFX96 PCR machine (Bio-Rad, Hercules, CA, USA), according to the manufacturers’ protocols. Gene expression was normalized to *β-actin* and expressed relative to the Young ZT0 group. Primer sequences are displayed in Supplementary Table 1. Several mice were excluded due to technical errors.

### Statistical analyses

*P* < 0.05 was considered statistically significant. Statistical analyses between groups were performed with unpaired *t*-tests or two-way ANOVA with post-hoc tests, where applicable. Šídák’s multiple-comparison test was used in experiment 1 to compare young vs. middle-aged mice within but not between diurnal phases. Tukey’s multiple-comparison test was used in experiment 2 to compare young vs. middle-aged mice within time points, as well as ZT0 vs. ZT12. Statistical analyses were performed with GraphPad Prism software, version 9.0.1 (GraphPad, La Jolla, CA, USA). Data are presented as means ± SD.

## Supplementary Materials

Supplementary Figures

Supplementary Table 1
