# Supplementary material for: Aging attenuates diurnal lipid uptake by brown adipose tissue
Source: Aging (Albany NY). 2022 Oct 4;14(19):7734–51. doi: 10.18632/aging.204318 (PMC9596214; doi:10.18632/aging.204318)
Supplement: Supplementary Figures [file aging-14-204318-s001.pdf]

SUPPLEMENTARY FIGURES

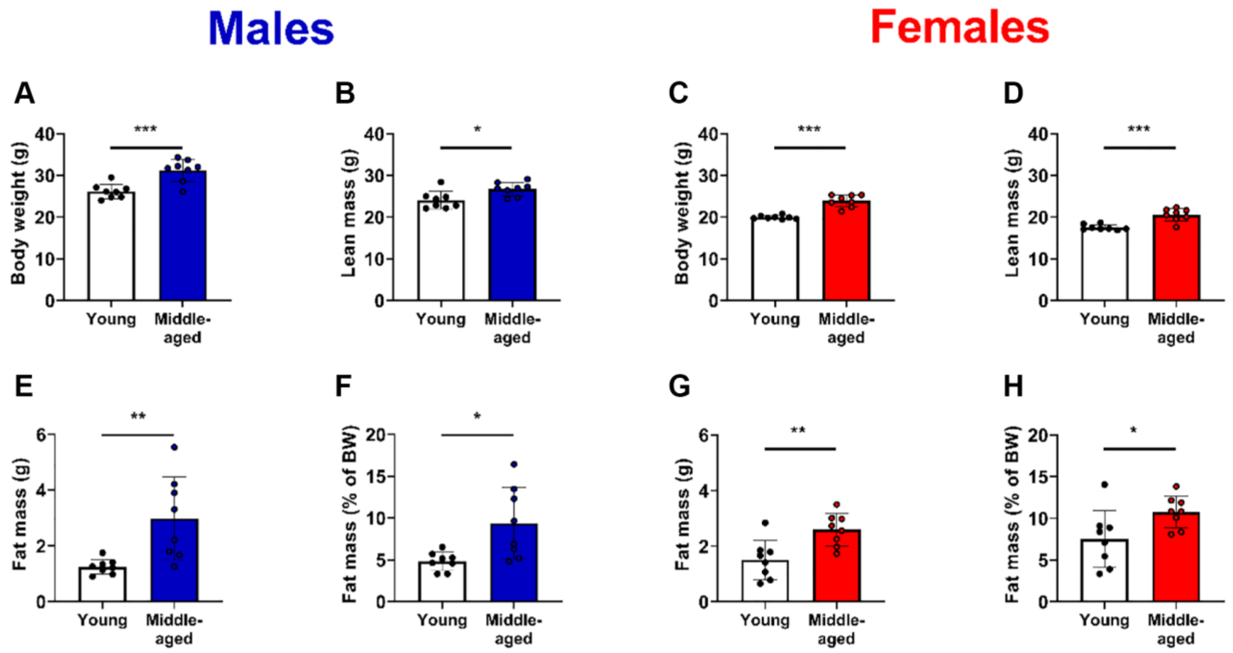

Supplementary Figure 1. Body composition and aging. Young (12 weeks old) and middle-aged (52 weeks old) male (left panels in blue) and female (right panels in red) C57BL/6J mice were compared (n = 8 mice/group). (**A**, **C**) body weight, (**B**, **D**) lean mass, and (**E**, **G**) fat mass from which (**F**, **H**) the percentage of fat mass relative to body weight (BW) was calculated. Bar graphs represent means ± SD. $^{*}p < 0.05$; $^{**}p < 0.01$; $^{***}p < 0.001$, according to unpaired t-test.

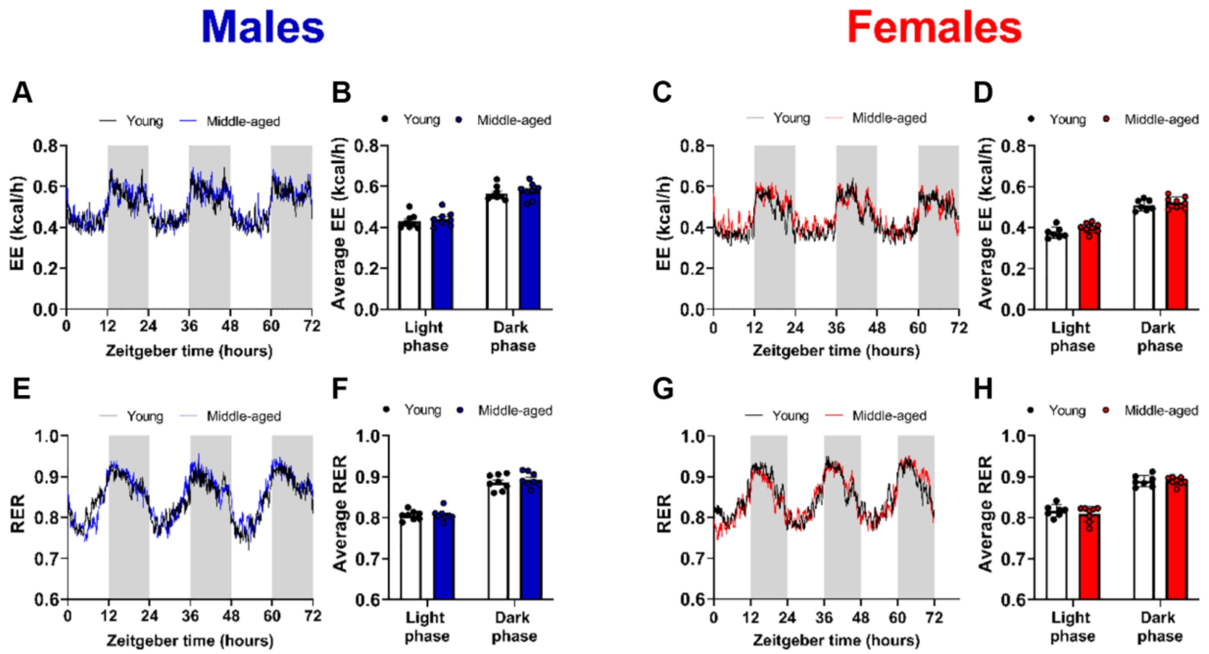

Supplementary Figure 2. Energy expenditure and respiratory exchange ratio during aging. Young (12 weeks old) and middle-aged (52 weeks old) male (left panels in blue) and female (right panels in red) C57BL/6J mice (n = 8 mice/group) were single-housed in metabolic cages for continuous measurements of O_2 consumption and CO_2 production, from which (**A**–**D**) energy expenditure (EE) and (**E**–**H**) respiratory exchange ratio (RER) were calculated. Bar graphs represent means ± SD. No significant differences were observed according to unpaired t-test and two-way ANOVA and following Šídák's multiple-comparison test.

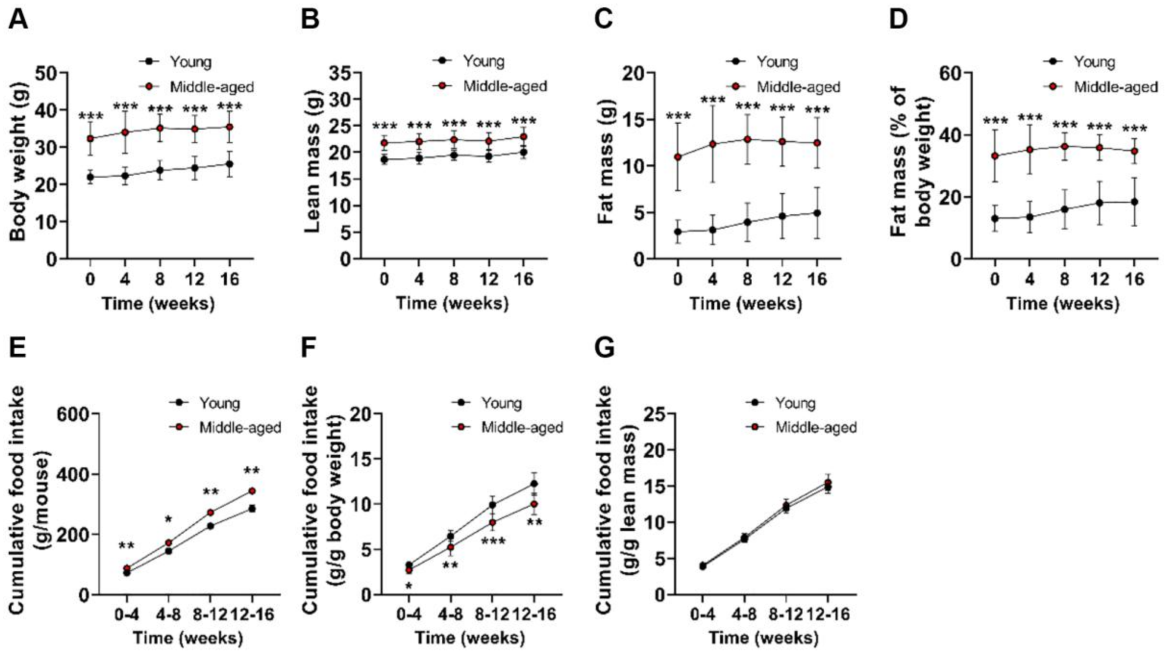

Supplementary Figure 3. Body composition and aging. Young (11–15 weeks old) and middle-aged (51–55 weeks old) female APOE*3-Leiden.CETP mice (n = 15–16 mice/group) were followed for 16 weeks to monitor (**A**) body weight, (**B**) fat mass, and (**C**) lean mass. (**D**) Fat mass was additionally expressed as a percentage of body weight. (**E**) Cumulative food intake per cage was calculated per 4 weeks, and corrected for (**F**) body weight and (**G**) lean mass (n = 16 mice/group). Data points on curves represent means ± SD. $^{*}p < 0.05$, $^{**}p < 0.01$, $^{***}p < 0.001$, according to two-way ANOVA and Tukey's multiple-comparison test.

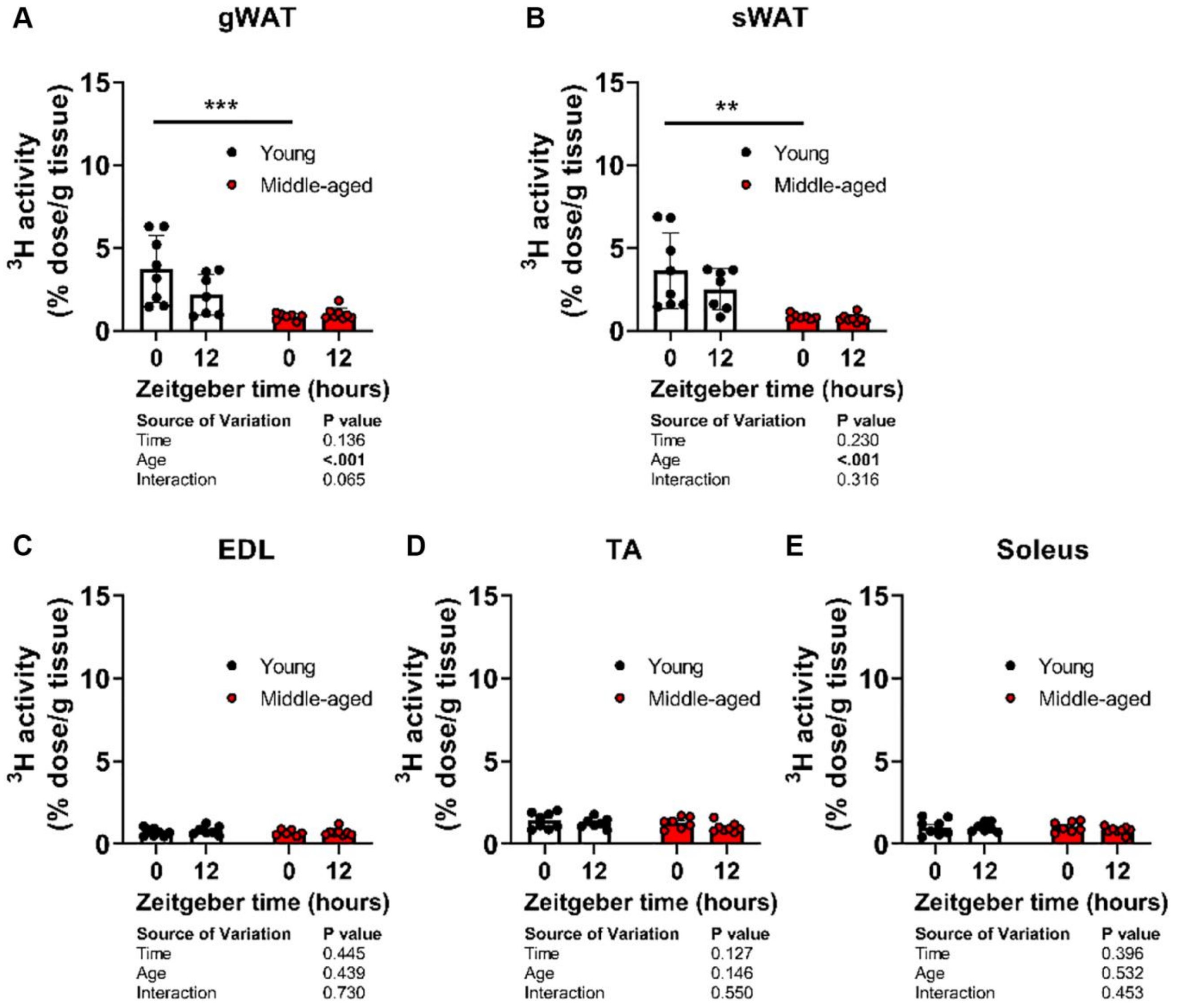

Supplementary Figure 4. Triglyceride-derived fatty acid uptake by white adipose tissues and skeletal muscles during aging. Young (27–31 weeks old) and middle-aged (67-71 weeks old) female APOE*3-Leiden.CETP mice were injected with triglyceride-rich lipoprotein-like particles double-labeled with glycerol tri[^{3}H]oleate and [^{14}C]cholesteryl oleate at *Zeitgeber* Time 0 and 12 (n = 6–8 mice/group/time point) to assess [^{3}H]oleate uptake by (**A**) gonadal white adipose tissue (gWAT), (**B**) subcutaneous WAT (sWAT), (**C**) extensor digitorum longus (EDL), (**D**) tibialis anterior (TA), and (**E**) soleus. Bar graphs represent means ± SD. $^{**}p < 0.01$; $^{***}p < 0.001$, according to two-way ANOVA and following Tukey's multiple-comparison test.
