# Supplementary material for: Aging attenuates diurnal lipid uptake by brown adipose tissue
Source: Aging (Albany NY). 2022 Oct 4;14(19):7734–51. doi: 10.18632/aging.204318 (PMC9596214; doi:10.18632/aging.204318)
Supplement: Supplementary Table 1 [file aging-14-204318-s002.pdf]

SUPPLEMENTARY TABLE

Supplementary Table 1. Primer list.

Gene	Primer sequence
Acc	Forward AGATGGCCGATCAGTACGTC
	Reverse GGGGACCTAGGAAAGCAATC
Acox	Forward TATGGGATCAGCCAGAAAGG
	Reverse ACAGAGCCAAGGGTCACATC
Angptl4	Forward GGAAAGAGGCTTCCCAAGAT
	Reverse TCCCAGGACTGGTTGAAGTC
β-actin	Forward AACCGTGAAAAGATGACCCAGAT
	Reverse CACAGCCTGGATGGCTACGTA
Bmal1	Forward ATGCCAAGACTGGACTTCCG
	Reverse TGCAGAAGCTTTTTCGATCTGC
Clock	Forward AGTTAGGGCTGAAAGACGGC
	Reverse GGTGTGGAGGAAGGGTCTGA
Cpt1	Forward GAGACTTCCAACGCATGACA
	Reverse ATGGGTTGGGGTGATGTAGA
Cry1	Forward AGAGGGCTAGGTCTTCTCGC
	Reverse GTGAGTCTGCTGACTGTCCC
Fasn	Forward GCGCTCCTCGCTTGTCGTCT
	Reverse TAGAGCCCAGCCTTCCATCTCCTG
Lpl	Forward CCCTAAGGACCCCTGAAGAC
	Reverse GGCCCGATACAACCAGTCTA
Lxra	Forward CTGCACGCCTACGTCTCCAT
	Reverse AAGTACGGAGGCTCACCAGCT
Nr1d1	Forward GTGCTTGTCTCTGCAGACCG
	Reverse TTGGTGAAGCGGGAAGTCTC
Per1	Forward ACGGCCAGGTGTCGTGATTA
	Reverse CCCTTCTAGGGGACCACTCA
Srebp1c	Forward AGCCGTGGTGAGAAGCGCAC
	Reverse ACACCAGGTCCTTCAGTGATTTGCT
Ucp1	Forward TCAGGATTGGCCTCTACGAC
	Reverse TGCATTCTGACCTTCACGAC
